# Multi-Sensitive Au NCs/5-FU@Carr-LA Composite Hydrogels for Targeted Multimodal Anti-Tumor Therapy

**DOI:** 10.3390/molecules29174051

**Published:** 2024-08-27

**Authors:** Chunxia Qi, Ang Li, Baoming Wu, Peisan Wang

**Affiliations:** 1 Department of Chemical and Pharmaceutical Engineering, Hefei Normal University, Hefei 230601, China; qicx666@mail.ustc.edu.cn; 2 School of Biomedical Engineering, Research and Engineering Center of Biomedical Materials, Anhui Medical University, Hefei 230032, China; la2013170076@163.com; 3 Inflammation and Immune Mediated Diseases Laboratory of Anhu Province, Hefei 230032, China

**Keywords:** gold nanoclusters, Carr, composite hydrogel, multi-modal antitumor therapy, photodynamic therapy, photothermal therapy, chemotherapy

## Abstract

Multifunctional targeted drug delivery systems have been explored as a novel cancer treatment strategy to overcome limitations of traditional chemotherapy. The combination of photodynamic therapy and chemotherapy has been shown to enhance efficacy, but the phototoxicity of traditional photosensitizers is a challenge. In this study, we prepared a multi-sensitive composite hydrogel containing gold nanoclusters (Au NCs) and the temperature-sensitive antitumor drug 5-fluorourac il (5-FU) using carboxymethyl cellulose (Carr) as a dual-functional template. Au NCs were synthesized using sodium borohydride as a reducing agent and potassium as a promoter. The resulting Au NCs were embedded in the Carr hydrogel, which was then conjugated with lactobionic acid (LA) as a targeting ligand. The resulting Au NCs/5-FU@Carr-LA composite hydrogel was used for synergistic photodynamic therapy (PDT), photothermal therapy (PTT), and chemotherapy. Au NCs/5-FU@Carr-LA releases the drug faster at pH 5.0 due to the acid sensitivity of the Carr polymer chain. In addition, at 50 °C, the release rate of Au NCs/5-FU@Carr-LA is 78.2%, indicating that the higher temperature generated by the photothermal effect is conducive to the degradation of Carr polymer chains. The Carr hydrogel stabilized the Au NCs and acted as a matrix for drug loading, and the LA ligand facilitated targeted delivery to tumor cells. The composite hydrogel exhibited excellent biocompatibility and synergistic antitumor efficacy, as demonstrated by in vitro and in vivo experiments. In addition, the hydrogel had thermal imaging capabilities, making it a promising multifunctional platform for targeted cancer therapy.

## 1. Introduction

Nowadays, malignant tumors have become the leading cause of global disease-related deaths, with people dying of cancer every day [1,2]. Conquering cancer has become an imperative issue for humanity. Although traditional chemotherapy methods have good pharmacological effects, they are limited by their poor stability, non-specific tumor-targeting ability, and serious toxic side effects [3,4]. To overcome these limitations, multifunctional targeted drug delivery systems have been widely explored as a novel cancer treatment strategy. Coordinated phototherapy and chemotherapy have demonstrated their ability to enhance treatment efficacy [5,6]. Photodynamic therapy (PDT) and photothermal therapy (PTT), two main types of phototherapy, rely on the heat or toxic reactive oxygen species (ROS) produced by the photosensitizer to kill tumor cells [7,8,9]. They have attracted widespread attention due to their non-invasive and controllable advantages. However, the phototoxicity of traditional photosensitizers is one of the main challenges for their potential clinical applications. The combination of PTT and PDT is an important strategy for cancer therapy, because many photosensitive materials have both photothermal conversion and singlet oxygen generation capabilities. For example, indoline green (ICG) is a clinical near-infrared fluorescent dye. It not only has strong photodynamic properties but also has certain photothermal properties and can be used for PDT and PTT. However, indoline green usually has poor stability and its bioavailability is insufficient, so its application is greatly limited. Therefore, compared with single therapy, combination therapy can achieve better tumor treatment effects.

In recent years, water-soluble gold nanoclusters (Au NCs) have been extensively investigated in medical diagnostic and therapeutic applications, such as bioanalysis, bioimaging, and cancer treatment, due to their small size, surface modifiability, and low toxicity [10,11,12,13,14]. One therapeutic application is the use of Au NCs as photosensitizers to generate highly reactive oxygen species clusters under light irradiation for tumor cell ablation. However, it is well-known that water-soluble nanoclusters suffer from poor stability and biocompatibility, which necessitates the development of nanoclusters with excellent phototherapeutic performance and minimal toxicity. This remains an urgent issue that needs to be addressed.

Local drug delivery at the tumor site is a highly promising approach that allows for precise delivery of drugs to specific areas, thereby minimizing damage to normal organs. Currently, several strategies, such as nanospheres, nanogels, and hydrogels, have been designed for local control of drug delivery and have demonstrated varying degrees of advantages [15,16,17,18,19,20]. Zhang et al. developed a Fe_3_O_4_@mSiO_2_-TPP/CDs nanocomposite by coupling carbon quantum dots synthesized with triphenylphosphine-modified Fe_3_O_4_@mSiO_2_ nanospheres for synergistic anti-tumor research with mitochondrial-targeting, long-lasting cell imaging and strong magnetic field cell uptake [21]. Among these drug storage systems, hydrogels have been proven to have less invasiveness, ease of management, and good biocompatibility. Their main feature is a hydrophilic polymer network that can expand to form a three-dimensional network structure in aqueous or physiological fluids [22,23]. Carrageenan (Carr), a polysaccharide material derived from red algae, can be effectively degraded under acidic and high-temperature conditions. When combined with a univalent cation, it can form a temperature- and pH-sensitive hydrogel, making it a promising candidate for drug delivery systems.

**Scheme 1:**
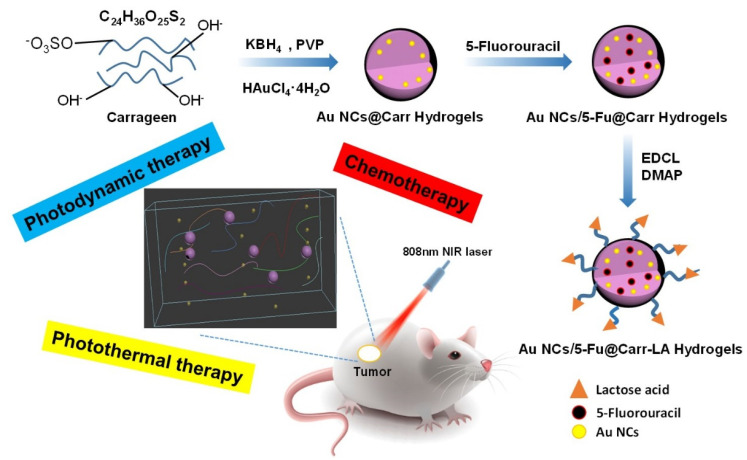
Potassium borohydride was used as a reducing agent of chloro-auric acid to prepare gold nanoclusters (Au NCs), and, at the same time, it was used as a potassium source to induce carrageenan to form a double-sensitive hydrogel, and nanoparticles containing Au NCs and the temperature-sensitive anticancer drug 5-flouoruracil (5-FU) were prepared. Au NCs/5-FU@Carr-LA nanogel particles can be used for the study of the synergistic anti-tumor effect of phototherapy and chemotherapy, which can specifically recognize the glycoprotein lactose acid molecule (LA) with galactose residue as a targeting agent.

In this study, we ingeniously used sodium borohydride not only as a reducing agent for chloroauric acid to prepare gold nanoclusters (Au NCs) but also as a potassium source to induce carrageenan to form a dual-responsive hydrogel, resulting in the formation of nanogels encapsulating Au NCs and the temperature-sensitive anticancer drug 5-fluorouracil (5-FU) (Scheme 1). These nanogels were externally functionalized with lactose-terminated glycoprotein lactalbumin (LA) as a targeting agent, and the resulting AuNCs/5-FU@Carr-LA nanogel particles were used for synergistic photodynamic and chemotherapy studies against cancer. In this system, the sulfate groups contained in the carrageenan hydrogel matrix can stabilize the water-soluble Au NCs. The Au NCs can serve as both photothermal agents and photosensitizers for photothermal and photodynamic therapy of tumors, respectively. Meanwhile, the elevated temperature induced by photothermal action can trigger the degradation of the carrageenan hydrogel matrix at the tumor site, releasing 5-FU for chemotherapy. In vitro and in vivo results demonstrate that the prepared sample has good synergistic PDT/PTT/chemotherapy anti-tumor effects and photothermal imaging capability. Such an integrated system with multiple sensitivities to light and the tumor microenvironment, high biocompatibility, excellent anti-tumor efficacy, and thermal imaging function is worth further exploration for more extensive applications.

## 2. Results and Discussion

**Figure 1:**
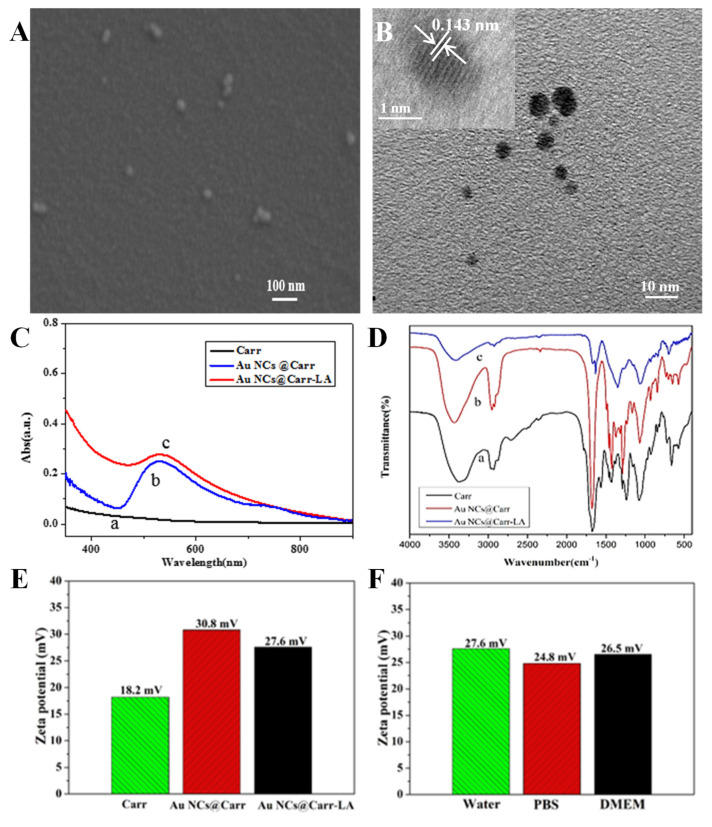
(**A**) The SEM image of Au NCs/5-FU@Carr-LA; (**B**) the TEM image of Au NCs. (The insets in (**B**) present the lattice spacing of Au NCs.) (**C**) UV-Vis-NIR spectra and (**D**) FTIR spectra of (a) Carr, (b) Au NCs@Carr, and (c) Au NCs@Carr-LA, respectively. (**E**) The zeta potential diagram of different samples in the medium. (**F**) Zeta potentials of different samples.

Figure 1A presents the scanning electron microscopy (SEM) image of AuNCs/5-FU@Carr-LA, indicating that the synthesized sample consists of spherical nanoparticles with uniform size and shape. The average size of the gel nanoparticles was approximately 60 nm. The internal structure of the nanoparticles can be observed from the transmission electron microscopy (TEM) image shown in Figure 1B, revealing the presence of Au NCs with a diameter of only about 3 nm and excellent monodispersity. Furthermore, the inset in Figure 1B showed a lattice fringe spacing of 0.141 nm corresponding to the (220) crystal plane of the Au NCs, demonstrating the successful synthesis of Au NCs in Carr. UV-Vis-NIR spectra of Carr, Au NCs@Carr, and Au NCs@Carr-LA were collected and shown in Figure 1C. Figure 1C-a displays the UV spectrum of Carr, indicating a distinct absorption peak at 230 nm. In Figure 1C-b, the absorption enhancement of Au NCs@Carr in the range of 500–600 nm was due to the in situ formation of gold nanoclusters in the carrageenan, which broadened its absorption in the visible-NIR range. The absorption curve of Au NCs@Carr-LA was modified upon the attachment of targeting molecule lactobionic acid to the exterior of gel nanoparticles, as demonstrated by the peak broadening in Figure 1C-c, confirming the successful modification of LA. These results demonstrated the successful synthesis of Au-Au NCs@Carr-LA with excellent NIR absorption properties, offering promising applications in photodynamic therapy research. Further verification of the sample composition was conducted through FTIR analysis. In Figure 1D-a, prominent absorption peaks were observed at around 1250 and 850 cm^−1^, which represent the sulfate group and the (1, 3)-β-D-galactose component, respectively [24,25]. The high and low absorptions at 1250 cm^−1^ indicate the presence of K-carrageenan as the main component. A strong absorption peak at 1540 cm^−1^ indicates a high protein content. Carrageenan showed an absorption peak at 805 cm^−1^ that was not present in other types [26]. In Figure 1D-b, the decrease in the absorption peak of the S-H bond at 2551 cm^−1^ indicates the interaction between the thiol ligand and Au NCs, indicating successful in situ synthesis of Au NCs coated with carrageenan without damaging the carrageenan structure [27]. The infrared spectrum of Au NCs@Carr-LA (Figure 1D-c) showed a significant enhancement in the intensity of two characteristic absorption peaks at 1610 and 1290 cm^−1^ compared to the previous two types of nanoparticles, indicating esterification between the hydroxyl group of lactose acid and the base on Carr [28]. The intensity of the characteristic peaks at 3400 and 1400 cm^−1^, which belong to -OH, also increased, mainly due to the presence of -OH in lactose acid molecules. These results demonstrated the successful preparation of Au NCs@Carr-LA. The stability of AuNCs/5-FU@Carr-LA nanogels was characterized using zeta potential (Figure 1E,F). The zeta potential of the nanocomposites significantly increased with the in situ generation of Au NCs, indicating improved stability. After LA modification, the zeta potential of AuNCs/5-FU@Carr-LA slightly decreased due to the negative charge carried by LA. Nevertheless, the nanogels still exhibited high stability, which is beneficial for their stable existence in various media.

Further characterization of the surface elemental composition of the Au NCs/5-FU@Carr-LA complex was investigated using XPS. The full spectrum in Figure S1A (in the Supplementary Materials) indicated the presence of six main elements: C, N, O, Au, S, and K. In Figure S1B (in the Supplementary Materials), the two peaks observed at 84.04 and 87.73 eV in the Au 4f region were assigned to the Au 4f 7/2 and Au 4f 5/2 electron states, respectively, confirming the presence of Au and providing evidence for the formation of Au-S bonds, which were further used for the synthesis of Au NCs [29]. The high-resolution S 2p spectrum in Figure S1C (in the Supplementary Materials) showed a peak at 163.28 eV corresponding to sulfate ions in carrageenan, which can interact with Au to form Au NCs [29,30]. The high-resolution K 2p spectrum in Figure S1D (in the Supplementary Materials) indicated the presence of K in carrageenan, with a binding energy of 292.88 eV, providing evidence for the formation of a hydrogel [31]. These results confirmed that the prepared nanocomposite material was composed of Au NCs connected to Carr and LA molecules.

**Figure 2:**
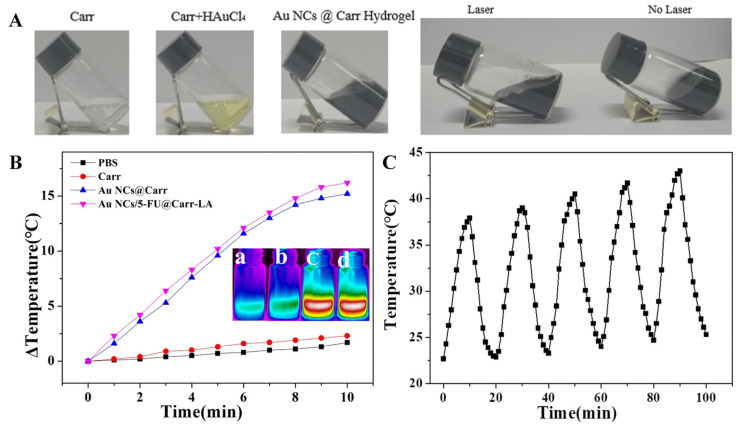
(**A**) The digital image of Au NCs@Carr hydrogel; sol-gel conversion of hydrogel. (**B**) Temperature curves of different samples irradiated with 808 nm laser at 1 W/cm^2^ for 10 min; the insets show the corresponding thermal images of PBS (a), Carr (b), Au NCs@Carr (c), and Au NCs/5-FU@Carr-LA (d). (**C**) The temperature change curve of Au NCs/5-FU@Carr-LA with irradiation for five cycles.

The formation of the hydrogel was demonstrated using the inversion test. As shown in Figure 2A, the sol-gel transition of the carrageenan solution and chloroauric acid solution resulted in the loss of fluidity and the formation of a semi-solid gel state, accompanied by a color change from light yellow to purple in the solution of the complex. Furthermore, to confirm the temperature sensitivity of the hydrogel, the hydrogel lost its gel state and regained fluidity when irradiated with an 808 nm laser, which provided the possibility for the subsequent temperature-triggered drug release. The light-thermal properties of the samples were analyzed. As shown in Figure 2B, under 808 nm near-infrared laser irradiation, the temperature of the control groups PBS and Carr only increased by 1.7 °C and 2.3 °C, respectively, within 10 min, indicating that Carr had no obvious photothermal properties. However, the temperature of the Au NCs@Car and Au NCs/5-FU@Carr-LA solutions increased by 15.2 °C and 16.2 °C, respectively, mainly due to the strong near-infrared absorption and excellent photothermal conversion performance of Au NCs. The temperature change curve of the Au NCs/5-FU@Carr-LA sample after five photo-irradiation cycles shows that Au NCs/5-FU@Carr-LA has high photothermal stability (Figure 2C). These results indicate that Au NCs/5-FU@Carr-LA is a promising photothermal agent with potential applications in photothermal therapy for tumors.

**Figure 3:**
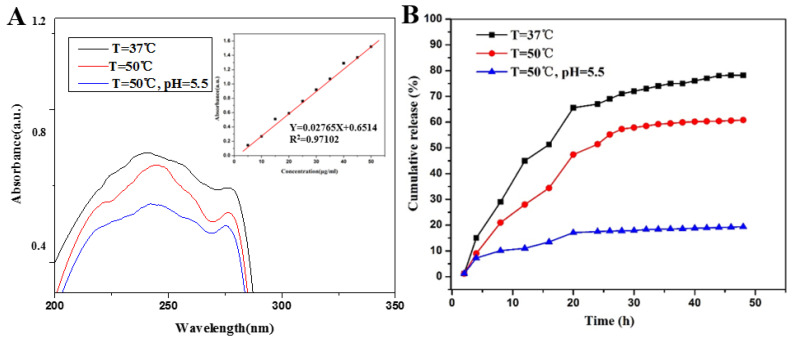
(**A**) UV-Vis spectra of Au NCs@Carr-LA loaded with 5-FU under different conditions. (The insets in (**A**) present the standard curve of the 5-FU.) (**B**) In vitro drug release profiles for the release of 5-FU from Au NCs/5-FU@Carr-LA in the different temperatures (37 °C, 50 °C) and pHs (5.0, 7.4) of the buffer solution.

UV-Vis spectroscopy was used to test the absorbance of 5-FU at 265 nm in a series of concentrations ranging from 5 to 50 µg/mL. The standard curve of 5-FU was obtained by processing the data, and the fitted equation of the curve was Y = 0.02765X + 0.6514, which exhibited good linearity (as shown in Figure 3A). Therefore, the absorbance changes of the solution before and after 5-FU was loaded onto the Au NCs@Carr-LA complex (as shown in Figure 3A) could be used to determine the drug-loading capacity of 5-FU in the complex, which was found to be 28.14 µg/mg. This indicated that the prepared composite gel nanoparticles had excellent drug-loading capacity. In order to verify the pH and temperature sensitivity of Carr, the release curves of 5-FU were measured at different pH values (5.0 and 7.4) and temperatures (37 and 50 °C). As shown in Figure 3B, Au NCs/5-FU@Carr-LA released the drug more rapidly at pH 5.0, mainly due to the high acid sensitivity of the Carr polymer chain in the shell of the composite gel nanoparticles, which could be easily hydrolyzed under acidic conditions, achieving the purpose of controlling drug release. Additionally, at a temperature of 50 °C, the release rate was higher, and the release rates of Au NCs/5-FU@Carr-LA were 78.2%, 58.7%, and 18.4%, respectively, indicating that the higher temperature generated by the photothermal effect was conducive to the degradation of the Carr polymer chain.

**Figure 4:**
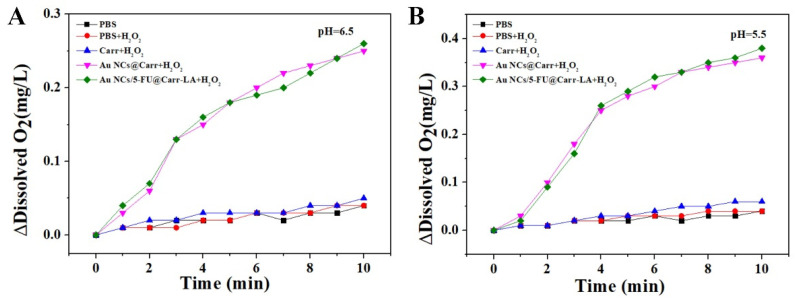
Changes of O_2_ generation with time from the decomposition of H_2_O_2_ catalyzed by different samples at pH (**A**) 6.5 and (**B**) 5.5.

The O_2_ generation under different pH conditions was measured using a portable oxygen meter within 10 min of catalyzing H_2_O_2_. As shown in Figure 4A, at pH 6.5, in the simulated tumor microenvironment (H_2_O_2_ concentration of 1 × 10^−4^ mol/L), Carr did not exhibit significant O_2_ production within 10 min compared to the control group PBS, indicating that Carr did not have the ability to catalyze H_2_O_2_ to produce O_2_. However, the maximum O_2_ production of the dispersed systems of Au NCs@Carr and Au NCs/5-FU@Carr-LA were 0.23 mg/L and 0.25 mg/L, respectively, indicating that both samples had some catalytic ability towards H_2_O_2_ to produce O_2_. As shown in Figure 4B, at pH 5.5, the O_2_ production ability of the Au NCs@Carr and Au Cs/5-FU@Carr-LA systems was more significant, with maximum O_2_ productions of 0.36 g/L and 0.38 g/L, respectively, indicating that acidic conditions were more conducive to catalyzing H_2_O_2_. These results suggested that AuNCs/5-FU@Carr-LA, which has the ability to catalyze H_2_O_2_ decomposition and produce O_2_, can alleviate the hypoxic environment of tumors and further improve PDT performance.

**Figure 5:**
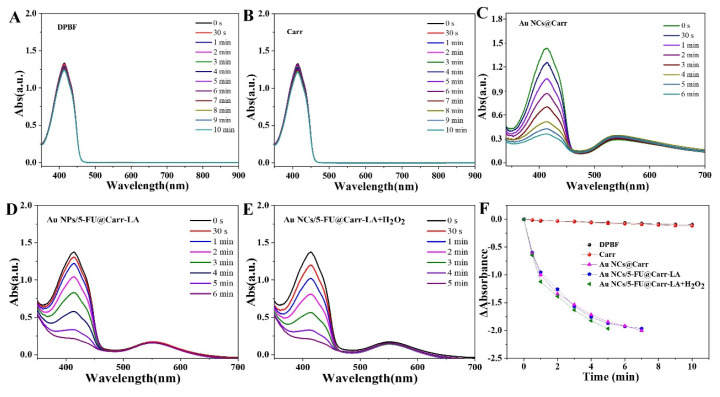
Photodynamic effects of (**A**) DPBF, (**B**) Carr, (**C**) Au NCs@Carr, and (**D**) Au NCs/5-FU@Carr-LA; (**E**) Au NCs/5-FU@Carr-LA+ H_2_O_2_; (**F**) the absorbance change curves of different sample dispersions at 410 nm.

The generation ability of singlet oxygen (^1^O_2_) induced by the sample under NIR light was investigated using the chemical probe DPBF. Figure 5A shows the UV-Vis absorption spectra of pure DPBF under 808 nm laser irradiation for different times. It can be seen that the characteristic peak of DPBF at 410 nm did not significantly decrease after 10 min, indicating that pure DPBF cannot be degraded under light. Similarly, the absorbance of the mixture of pure Carr and DPBF did not decrease after 10 min of irradiation (Figure 5B), indicating that Carr did not generate ^1^O_2_. In contrast, the absorbance of Au NCs@Carr and DPBF decreased significantly after 6 min of light irradiation (Figure 5C), indicating that Au NCs@Carr can generate ^1^O_2_. The DPBF absorbance curve of AuNCs/5-FU@Carr-LA also decreased to almost the lowest level after 6 min (Figure 5D), indicating that the drug 5-FU and co-surfactant LA do not affect the ability of Au NCs/5-FU@Carr-LA to generate ^1^O_2_. Figure 5E records the relative absorbance change of DPBF in different sample solutions, and it can be seen that the absorbance in the Au NCs/5-FU@Carr-LA solution decreased rapidly, further verifying the excellent PDT performance of Au NCs/5-FU@Carr-LA.

Fluorescence microscopy was used to observe the endocytosis of Au NCs/5-FU@Carr-LA in HepG2 cells. No green fluorescence was observed in the images of FITC-dextran co-cultured with cells (Figure S2A in the Supplementary Materials), indicating that no samples were internalized by the cells. In Figure S2B (in the Supplementary Materials), FITC-dextran-labeled Au NCs/5-FU@Carr showed weak green fluorescence, indicating that a small amount of the product was internalized by HepG2 cells. In contrast, significant green fluorescence was observed in the Au NCs/5-FU@Carr-LA group (Figure S2C in the Supplementary Materials), indicating that Au NCs/5-FU@Carr-LA was endocytosed by HepG2 cells. These results demonstrated that the targeting ability of LA promotes the endocytosis of AuNCs/5-FU@Carr-LA by HepG2 cells.

**Figure 6:**
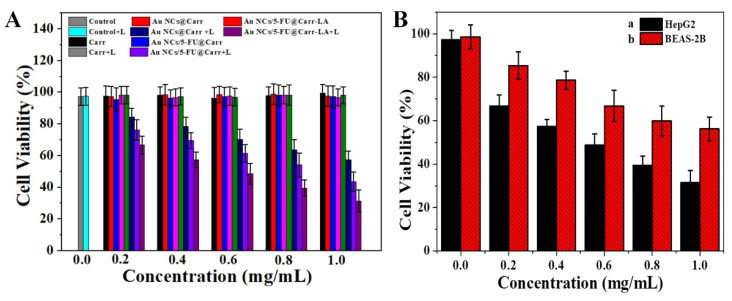
(**A**) Viability of HepG2 cells incubated with different concentrations of samples under different conditions, respectively; (**B**) cytotoxicity assays of (a) HepG2 and (b) BEAS-2B cells incubated with various concentrations of Au NCs/5-FU@Carr-LA with laser irradiation.

In order to investigate the cellular toxicity of AuNCs/5-FU@Carr-LA, HepG2 cells were incubated with different concentrations of AuNCs/5-FU@Carr-LA for 24 h to detect cell viability. As shown in Figure 6A, the viability of all groups under dark conditions was above 96%, indicating good biocompatibility of the samples. Under 808 nm laser irradiation, the survival rate of Carr group cells did not decrease significantly with increasing sample concentration, indicating that Carr also has excellent biocompatibility. The cell survival rates of the Au NCs@Carr light group were 84.4%, 78.5%, 70.2%, 63.6%, and 7.4% at respective sample concentrations. Meanwhile, the Au NCs/5-FU@Carr light group had survival rates of 75.2%, 69.5%, 61.4%, 54.2%, and 43.5%, respectively, which was due to the synergistic effect of Au NCs and 5-FU inducing cell death. The corresponding cell viabilities of the Au NCs/5-FU@Carr-LA light group at the same concentrations were 66.7%, 57.4%, 48.7%, 39.4%, and 31.5%, indicating that LA can target tumor cells and enhance the synergistic effect of PTT/PDT/chemotherapy. Furthermore, HepG2 cells and normal cells BEAS-2B were used as a comparison to verify the targeting effect of LA on cell-killing efficacy. As shown in Figure 6B, due to the targeting effect of LA on HepG2 cells, the cell survival rate of Au NCs/5-FU@Carr-LA decreased continuously with increasing concentration under light conditions and was significantly better than the effect on normal cells. These results indicate that Au NCs/5-FU@Carr-LA can cure tumors through a combination of PTT/PDT/chemotherapy.

**Figure 7:**
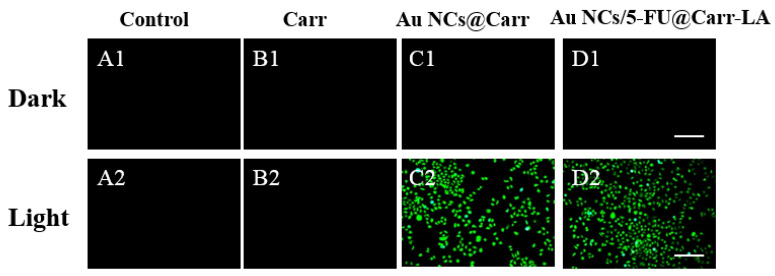
Fluorescence microscopy images of HepG2 cells incubated with (**A1**,**A2**) control groups (without samples), (**B1**,**B2**) Carr, (**C1**,**C2**) Au NCs@Carr, (**D1**,**D2**) Au NCs/5-FU@Carr-LA, (**A1**,**B1**,**C1**,**D1**) without laser irradiation, and (**A2**,**B2**,**C2**,**D2**) with laser irradiation (808 nm, 1 W/cm^2^), respectively.

To investigate the ability of AuNCs/5-FU@Carr-LA to generate ^1^O_2_, DCFH-DA was used as a probe. Under dark conditions, no green fluorescence was observed in any of the groups (Figure 7(A1–D1)), indicating that ROS could not be generated in the absence of light. Under light conditions, no green fluorescence was observed in the control group (Figure 7(A2)), indicating that laser irradiation by itself cannot induce the generation of ^1^O_2_. The Carr group showed very little green fluorescence (Figure 7(B2)), indicating that Carr had no ability to produce ROS. In contrast, the Au NPs@Carr light group showed significant green fluorescence (Figure 7(C2)), indicating the generation of a large amount of ROS. Similarly, the fluorescence intensity of the Au NCs/5-FU@Carr-LA light group was comparable to that of the AuNCs@Carr light group (Figure 7(D2)), indicating that Au NCs/5-FU@Carr-LA can serve as a good photosensitizer for PDT-based anti-tumor therapy.

In order to visualize the distribution of live and dead cells, HepG2 cells were co-cultured with different samples and stained with Hoechst and PI solutions, which were used to stain live and dead cells as blue and red, respectively. Under dark conditions, all groups exhibited blue fluorescence (Figure S3A–E, in the Supplementary Materials), indicating no obvious damage to HepG2 cells. Under light conditions, the control group showed no red fluorescence (Figure S3(F3), in the Supplementary Materials), indicating that cell death was not induced by laser irradiation alone. Similarly, no obvious red fluorescence was observed in the Carr group (Figure S3(G3), in the Supplementary Materials), demonstrating good biocompatibility of the Carr sample. A small amount of red fluorescence was observed in the fitting graph of the Au NCs@Carr group (Figure S3(H3) in the Supplementary Materials), indicating some cytotoxicity of Au NCs@Carr to cells. When 5-FU was added, the red fluorescence in the Au NCs/5-FU@Carr light group significantly increased (Figure S3(I3) in the Supplementary Materials), which was due to the combined killing effect of the 5-FU chemotherapy drug and AuNCs@Carr. Addition of LA led to almost complete cell death in the AuNCs/5-FU@Carr-LA light group (Figure S3(J3) in the Supplementary Materials). These results were consistent with the MTT assay results (Figure 6), further demonstrating the excellent anti-tumor performance of AuNCs/5-FU@Carr-LA.

**Figure 8:**
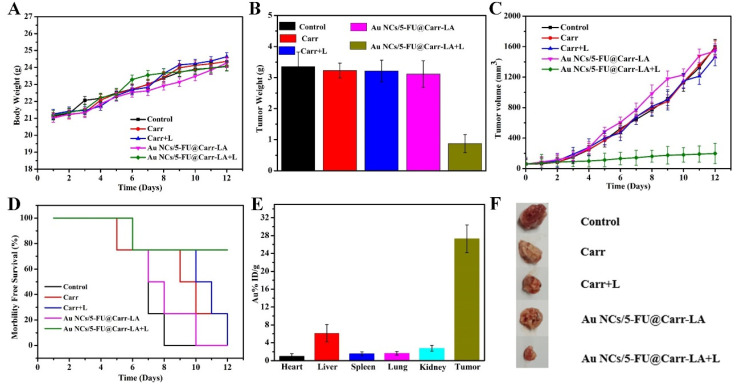
In vivo antitumor effect of samples under different conditions. (**A**) Time-dependent body weight change curves, (**B**) the tumor weight change histograms, (**C**) tumor-volume curves, and (**D**) the survival rates of the ICR mice treated with different samples. (**E**) Biodistribution of Au in main organs and tumor after intravenous injection of Au NCs/5-FU@Carr-LA for 12 h. (**F**) The physical image of the tumor (L represents laser).

Five groups of ICR mice were used to study the in vivo anticancer effect of Au NCs/5-FU@Car-LA. As shown in Figure 8A, there was a slight variation in the body weight of each mouse, indicating a high level of biocompatibility and biosafety of Au NCs/5-FU@Carr-LA in vivo. The anticancer efficacy was verified by measuring the tumor mass and volume of the mice (Figure 8B,C). After 12 days, the group treated with Au NCs/5-FU@Carr-LA under light irradiation showed the minimum tumor mass and volume, which was the result of the synergistic effect of PDT and chemotherapy. The survival rate of ICR mice after treatment was calculated for each group (Figure 8D). The control group died successively within eight days. Compared with the control group, the other three groups showed a certain degree of slowing down of the mice death rate after treatment, but not enough to indicate that the tumor cells had been killed. The survival rate of mice treated with Au NCs/5-FU@Carr-LA+L was significantly higher than that of all other groups, with a relative survival rate of 80% after NIR laser irradiation at 808 nm due to the targeting of LA to cancer cells and the synergistic effect of PDT/PTT/chemotherapy, indicating a significant anticancer effect. Figure 8E shows the main distribution of Au elements in various organs and tumors, with the highest concentration of Au elements in the tumor, indicating that the samples can accumulate well in the tumor site for cancer treatment. Similarly, the tumor sites of the mice in each group were dissected for comparison (Figure 8F), and Au NCs/5-FU@Carr-LA+L showed the best anticancer effect.

In order to assess the suitability of the nanoplatform for in vivo applications, serum compatibility is a critical indicator. To further validate the stability and biocompatibility of the nanoplatform, a red blood cell hemolysis assay was designed. In the experiment (Figure S4A in the Supplementary Materials), a bright red color was observed in the pure water control group, indicating that the red blood cells were ruptured. In contrast, no obvious hemolysis was observed in the PBS and Au NCs/5-FU@Carr-LA groups. When the concentration of the nanoplatform was changed from 200 μg/mL to 1200 μg/mL, the hemolysis percentage remained below 5% (Figure S4B, in the supporting information), indicating that it would not cause red blood cell rupture. Therefore, it can be concluded that the nanoplatform has excellent stability for intravenous administration and can be used in the bloodstream system.

Each group of mice was dissected to verify the effect of the treatment on normal tissue organs. As shown in Figure S5 (in the Supplementary Materials), after 12 days of treatment, heart, liver, spleen, lung, and kidney tissue organs were stained with H&E. The H&E staining images of the experimental group were compared with those of the blank control group, and no obvious organ damage or inflammatory lesions were observed in the hearts, livers, spleens, lungs, or kidneys of the mice. In addition, after 40 days, the treated mice were dissected, and their tissue organs were stained. The results showed that Au NCs/5-FU@Carr-LA had good in vivo biocompatibility, and its long-term toxicity evaluation also indicated no toxic side effects on normal tissue organs, which suggests its potential for future clinical research. Au NCs/5-FU@Carr-LA demonstrated excellent photothermal properties, which may be promising for in vivo photothermal imaging. As shown in Figure S6 (in the Supplementary Materials), after 10 min of 808 nm laser irradiation, the injection of Au NCs/5-FU@Carr-LA into the mouse body rapidly increased the temperature by 17 °C, while the injection of PBS as the control group showed only a slight increase in temperature, rising by only 2 °C. This indicates that Au NCs/5-FU@Carr-LA can be used for photothermal imaging, providing a potential tool for identifying tumors and normal tissues.

## 3. Materials and Methods

### 3.1. In Situ Synthesis of AuNCs@Carr Hydrogel

An amount of 0.2505 g of Carr (Aladdin Reagent Co., Ltd. (Shanghai, China)) was dissolved in 40 mL of deionized water to prepare a solution with a concentration of 6.25 mg/mL under magnetic stirring. Then, 0.25 g of KBH_4_ (Aladdin Reagent Co., Ltd. (Shanghai, China)) with different volumes was added to 10 mL of deionized water, mixed and ground for 30 min, and stirred at room temperature for 5 h.

To synthesize Au NCs@Carr hydrogel, 0.35 mL of HAuCl_4_ (Aladdin Reagent Co., Ltd. (Shanghai, China)) water solution (40 mM) and 2 mL of Carr (6.25 mg/mL) were mixed thoroughly and sonicated for 30 min. Subsequently, 0.2 mL of KBH_4_ solution (25 mg/mL) was added dropwise to the composite solution to observe the color change of the solution and then stirred for 4 h under magnetic stirring. Finally, the product was centrifuged and freeze-dried to obtain Au NCs@Carr nano-hydrogel particles.

### 3.2. Synthesis of Au NCs/5-FU@Carr Composite Hydrogel Particles

An amount of 0.35 mL of HAuCl_4_ water solution (40 mM) was mixed with 4 mL of Carr solution (6.25 mg/mL), followed by sonication for 30 min. Then, 0.2 mL of KBH_4_ solution (25 mg/mL) was added dropwise to the composite solution, and 4 mL of PVP (Aladdin Reagent Co., Ltd. (Shanghai, China)) solution (5.3 mg/mL) was added immediately. The mixture was stirred magnetically for 12 h. A 5 mg/mL 5-FU (Aladdin Reagent Co., Ltd. (Shanghai, China)) solution was prepared and mixed thoroughly in a 1:7 volume ratio. After repeated washing, the composite hydrogel small particle powder was obtained by freeze-drying.

### 3.3. Synthesis of Au NCs/5-FU@Carr-LA Composite Hydrogel Particles

Amounts of 0.12 g of LA (Aladdin Reagent Co., Ltd. (Shanghai, China)), 0.0326 g of EDC (Aladdin Reagent Co., Ltd. (Shanghai, China)), and 0.0239 g of DMAP (Aladdin Reagent Co., Ltd. (Shanghai, China)) were mixed and added to 10 mL of water. The mixture was stirred in a 37 °C water bath for 2 h for full activation, referred to as solution A. An amount of 0.05 g of previously prepared Au NCs/5-FU@Carr solution was dissolved in 5 mL of water to prepare 5 mL of a solution B with a concentration of 10 mg/mL. Solution A was slowly added to solution B, and the pH was adjusted to 8.5 using a 0.1 M NaOH (Aladdin Reagent Co., Ltd. (Shanghai, China)) solution. The mixture was stirred vigorously for 24 h, and the resulting solution was collected, centrifuged, and washed three times each with ethanol and ultrapure water. The product was then freeze-dried to obtain the Au NCs/5-FU@Carr-LA composite hydrogel nanoparticles.

### 3.4. Photothermal Conversion Capability

Samples including Carr, Au NCs@Carr, Au NCs/5-Fu@Carr, and Au NCs/5-Fu@Carr-LA were dispersed in PBS at a concentration of 1 g/mL. PBS was used as the control group. The temperature changes of each sample were recorded using a thermal imaging camera at intervals of 1 min under laser irradiation.

### 3.5. Detection of Catalytic Oxygen Production Ability

The catalytic oxygen production ability of the samples was determined using a portable dissolved oxygen meter. Carr, Au NCs@Carr, Au NCs/5-FU@Carr, and Au NCs/5-FU@Carr-LA (concentration of 1 mg/mL) were added to 20 mL of PBS solution containing 10^4^ mol/L H_2_O_2_ (Aladdin Reagent Co., Ltd. (Shanghai, China)), and the same amounts of the samples were added to the PBS solution without 10^4^ mol/L H_2_O_2_ as the control group. The dissolved oxygen content of each sample was recorded using a portable dissolved oxygen meter at pH 6.5 and 5.5 for 10 min.

### 3.6. Drug Loading and Release

Drug loading estimation: At room temperature, 1 mL of 5-FU (1.0 mg/mL) was added to the dispersion of Au NCs@Carr in PBS (5 mL, 10 mg) and sonicated for 3 min. After stirring for one day, the upper clear liquid was collected by centrifugation and diluted at different concentrations, and the absorbance change at 265 nm was measured. The drug loading was calculated using Formula (1). The above procedure was repeated three times to obtain the average value. The estimated concentration of 5-FU in the nanocarrier was determined by analyzing the standard calibration curve obtained by analyzing the known concentrations of 5-FU solutions at 295 nm. The drug encapsulation efficiency was calculated using the following formula:

(1) $$Encapsulation\;efficiency=\frac{m_{1}-m_{2}}{m}*100\%$$

where m_1_ is the initial amount of the drug, m_2_ is the amount of the drug present in the supernatant, and m is the total amount of the drug.

To investigate the drug release, the release of 5-FU from Au NCs/5-FU@Carr-LA nanocarriers was monitored in PBS solution at two different pH conditions (pH 7.4 and 5.5) using the dialysis membrane method. For the drug release study, 2 g/mL of the nanocarrier was placed in a dialysis bag and immersed in 10 mL of PBS at 37 °C. At regular time intervals, 2 mL aliquots were collected and replaced with an equal volume of PBS to maintain sink conditions. The collected aliquots were analyzed by UV-Vis spectroscopy at 265 nm. Finally, the cumulative percentage of 5-FU release was plotted against time, and the release was calculated using the following equation:

(2) $$Cumulative\;release(\%)=\frac{amount\;of\;released\;drug}{amount\;of\;loaded\;drug}*100\%$$

Release of the 5-FU drug in the Au NCs/5-FU@Carr system: At room temperature, 10 mg of the Au NCs/5-FU@Carr sample was dispersed in 50 mL of PBS buffer solution under different pH conditions, and the supernatant was collected at regular intervals to measure its absorbance value. The release of 5-FU from the Au NCs/5-FU@Carr system in the PBS buffer solution at different pH values was calculated using the 5-FU standard curve.

### 3.7. Detection of Singlet Oxygen (^1^O_2_)

Singlet oxygen (^1^O_2_) was detected using a solution of 5 g/mL DPBF (Tianjin Chemical Co., Ltd. (Tianjin, China)), which undergoes a change in absorbance at 410 nm upon reaction with ^1^O_2_. The different samples were mixed with DPBF to obtain a homogeneous solution, and the absorbance changes were recorded under laser irradiation.

### 3.8. Detection of Intracellular ROS

Intracellular reactive oxygen species (ROS) were observed using 2′,7′-dichlorofluorescein diacetate (DCFH-DA) (Tianjin Chemical Co., Ltd. (China)). HepG2 cells were seeded in a 24-well plate for 24 h and washed three times with PBS before adding different samples for continued incubation for 8 h. All groups were co-incubated with 10 μmol/L of DCFH-DA for 50 min, washed three times with buffer, and then observed under a microscope after 3 min of irradiation with an 808 nm laser.

### 3.9. Cell Viability (MTT) Assay

Cell viability was assessed using the MTT (Tianjin Chemical Co., Ltd. (China)) assay. Tumor cells and normal cells were seeded into a 24-well plate and cultured for 24 h. After washing three times with PBS, cells were treated with different concentrations of samples (0, 0.2, 0.4, 0.6, 0.8, and 1 mg/mL) in 200 μL DMEM (Tianjin Chemical Co., Ltd. (China)) for 24 h. The light exposure group was subjected to laser irradiation for 10 min before continuing with the culture. Afterwards, the nutrient-depleted medium was removed and replaced with 100 μL of fresh DMEM containing 20 μL MTT. The cells were further cultured for 4 h, followed by the addition of 100 μL DMSO to dissolve the produced crystals. The absorbance was measured using a microplate reader.

### 3.10. Double Staining Fluorescence Assay

To further investigate the cytotoxicity of the samples, the viability of HepG2 cells was evaluated using calcein-AM and PI staining. DMEM dispersion containing different samples (300 μL) was added to each well, and the plate was incubated in the dark for 24 h. After 4 h of culture, the light exposure group was subjected to 10 min of 808 nm laser irradiation. All groups were further cultured for 24 h, and the culture medium was replaced with PBS. The cells were then stained with 0.5 mL calcein-AM (10 μg/mL) (Tianjin Chemical Co., Ltd. (China)) and 0.5 mL PI (10 μg/mL) (Tianjin Chemical Co., Ltd. (China)) for 15 min. The cells were visualized using a microscope.

### 3.11. Cell Phagocytosis Assay

First, HepG2 cells of logarithmic growth were inoculated in a confocal culture dish (2 mL, 1 × 10^5^ cells/dish), cultured in an incubator for 24 h, and then the culture medium was removed. A FITC-dextran probe was used to label the cellular uptake of samples. Au NCs/5-FU@Carr and AuNCs/5-FU@Carr-LA (1 mg/mL) were added to the culture medium containing FITC-dextran and incubated for 24 h. After washing away the nutrient-deprived medium with the buffer, incubated with HepG2 cells in a confocal culture dish for 24 h, observation of the cellular uptake was carried out under a microscope.

### 3.12. In Vivo Synergistic Antitumor Effect

In vivo photothermal imaging: All animal experiments were conducted according to national guidelines. H22 cells (mouse liver cancer cells) were subcutaneously transplanted into mice, and when the tumor volume reached about 1000 mm^3^, different sample dispersion solutions were injected into the mice. Four hours later, time-lapse photothermal images were taken every 1 min to evaluate the photothermal imaging ability of Au NCs/5-Fu@Carr-LA. Anti-tumor effect: ICR mice were randomly divided into groups, and different samples were intravenously injected into the mice. The mice’s body weights and tumor volumes were measured at fixed intervals, and the anti-tumor effect of the samples was evaluated by H&E staining of tumor tissues.

## 4. Conclusions

In this study, a novel dual-mode tumor therapeutic agent combining optical therapy and chemotherapy was designed. The agent was prepared using carrageenan extracted from red algae as a raw material, potassium borohydride as a coagulant and reducing agent, and chloroauric acid aqueous solution in situ reduced to form Au NCs, which were uniformly encapsulated in a natural water gel three-dimensional network. The gel was loaded with the temperature-sensitive drug (5-FU) and conjugated with the targeting molecule LA, resulting in AuNCs/5-FU@Carr-LA composite hydrogel particles. The hydrogel degrades under weakly acidic and heated conditions, releasing 5-FU for chemotherapy. In addition, the photothermal imaging properties of the gold nanoclusters were utilized to achieve a diagnosis-therapy integrated treatment mode. In vitro and in vivo experiments demonstrated excellent tumor tissue killing effects and high biocompatibility. This natural green treatment method integrating targeting, chemotherapy, and light therapy provides a new idea for tumor treatment and is worth further development and exploration of a wider range of applications. However, the structure-activity relationship of water-soluble gold clusters needs further study. Other physiological characteristics of the tumor microenvironment can also be considered, and the corresponding new nano platform can be designed for anti-tumor research in the future.

## Data Availability

The original contributions presented in the study are included in the article/Supplementary Material, further inquiries can be directed to the corresponding author.

## Supplementary Materials

The following supporting information can be downloaded at https://www.mdpi.com/article/10.3390/molecules29174051/s1: Figure S1: (A) XPS survey spectra of the as synthesized Au NCs/5-FU@Carr-LA; the high-resolution XPS spectra of (B) Au 4f; (C) S 2p; (D) K 2p; Figure S2: CLSM images of FITC-dextran labeled (A) HepG2 cells, (B) Au NCs/5-FU@Carr and (C) Au NCs/5-FU@Carr-LA; Figure S3: Fluorescence images of HepG2 cells incubated with (A,F) Control (without samples), (B,G) Carr, (C,H) Au NCs@Carr, (D,I) Au NCs/5-FU@Carr, (E,J) Au NCs/5-FU@Carr-LA without laser irradiation (A–E) or with laser irradiation (F–J); Figure S4: (A) The hemolysis percentage in the presence of the Au NCs/5-FU@Carr-LA at different concentrations and (B) The hemolysis photographs; Figure S5: The H&E staining of mice main organs after Au NCs/5-FU@Carr-LA treatments for 12 and 40 days. (Scale bar: 200 μm); Figure S6: The corresponding IR images of ICR tumor-bearing mice treated with control (top) and Au NCs/5-FU@Carr-LA (bottom) under irradiation at varied time intervals (0, 2, 4, 6, 8, and 10 min).
