# Supplementary material for: Multi-Sensitive Au NCs/5-FU@Carr-LA Composite Hydrogels for Targeted Multimodal Anti-Tumor Therapy
Source: Molecules. 2024 Aug 27;29(17):4051. doi: 10.3390/molecules29174051 (PMC11397649; doi:10.3390/molecules29174051)
Supplement: Supplementary file 1 [file molecules-29-04051-s001.zip › molecules-3137032-supplementary.pdf]

Supporting Information

Multi-Sensitive Au NCs/5-FU@Carr-LA Composite Hydrogels for Targeted Multimodal Anti-Tumor Therapy

Chunxia Qi [1,†], Ang Li [2,†], Baoming Wu [3,*] and Peisan Wang [2,*]

[1] Department of Chemical and Pharmaceutical Engineering, Hefei Normal University, Hefei 230601, China; qicx666@mail.ustc.edu.cn
[2] School of Biomedical Engineering, Research and Engineering Center of Biomedical Materials,
Anhui Medical University, Hefei 230032, China; la2013170076@163.com
[3] Inflammation and Immune Mediated Diseases Laboratory of Anhu Province, Hefei 230032, China
* Correspondence: wubaoming@ahmu.edu.cn (B.W.); wangpeisan@ahmu.edu.cn (P.W.)
† These authors contributed equally to this work.

Materials and methods

Materials

Potassium borohydride (KBH_4), 5-fluorouracil (5-FU), methylene dichloride (CH_2Cl_2), polyvinylpyrrolidone (PVP), 1-(3-dimethylamino-propyl) -3-ethylcarbon diimide (EDC), 4-dimethylaminopyridine (DMAP), N, n-dimethylformamide (DMF), sodium hydroxide (NaOH), lactobionic acid (LA), κ-carrageenan (κ-Carr), gold chloride tetrahydrate ($HAuCl_4 \cdot 4H_2O$), hydrochloric acid (HCl), hydrogen peroxide (H_2O_2), Glutathione (GSH, reduced) ($C_{10}H_{17}N_3O_6S$) were obtained from Aladdin Reagent Co., Ltd. (Shanghai, P. R. China). Dulbecco's Modified Eagle's Medium (DMEM), fetal calf serum, trypsin-EDTA solution, 3-[4, 5-dimethylthialzol-2-yl]-2, 5-diphenyltetrazolium bromide (MTT), Diphenylbenzofuran (DPBF), Hoechst 33342, propidium iodide (PI) were purchased from Tianjin Chemical Co., Ltd. (P. R. China). HepG2 cells and BEAS-2B cells were obtained from the Key Laboratory of Ecological Engineering and Biotechnology of Anhui Province, School of Life Science, Anhui University (Hefei, P. R. China). Female ICR mice were purchased from the Laboratory Animal Center of AnHui Medical University. All chemical reagents used in this experiment were of analytical grade without further purification. Deionized (DI) water is obtained from Millipore Milli-Q system (resistivity: 18.2 MΩ.cm).

Animal Models

Mice were obtained from the Laboratory Animal Center of Anhui Medical University (Certification of quality 34000200000077, 34000200000078). This study was conducted in strict accordance with the recommendations in the Regulations on the Management of Laboratory Animals in China promulgated in 1988. All the animal experiments were in agreement with the guidelines of the Animal Experiments and Care Regulations of Anhui University and approved by the Animal Use Committee of Anhui University.

Characterization

Transmission electron microscopy (TEM) images were obtained using a JEM-100SX instrument. Scanning electron microscope (SEM) was performed on an S-4800 (Hitachi

Co., Ltd., Japan) scanning electron microscope. Ultraviolet Visible (UV-Vis) measurements were performed by an UV-3900 spectrometer (Hitachi Co., Ltd., Japan). X-ray diffraction (XRD) patterns were recorded using a DX-2700 diffractometer (Dandong Fangyuan Instrument Co. Ltd., P. R. China). A superconducting quantum interference device (SQUID) magnetometer (Quantum Design PPMS XL-7) was used to measure the magnetic properties of the as-prepared samples. The OD values of the MTT assay were measured by a RT-2100C spectro-photometric micro-plate reader (Rayto, Shenzhen, P. R. China). Fluorescence microscopy images were obtained using a DMI3000B inverted fluorescence microscope (Leica, Germany).

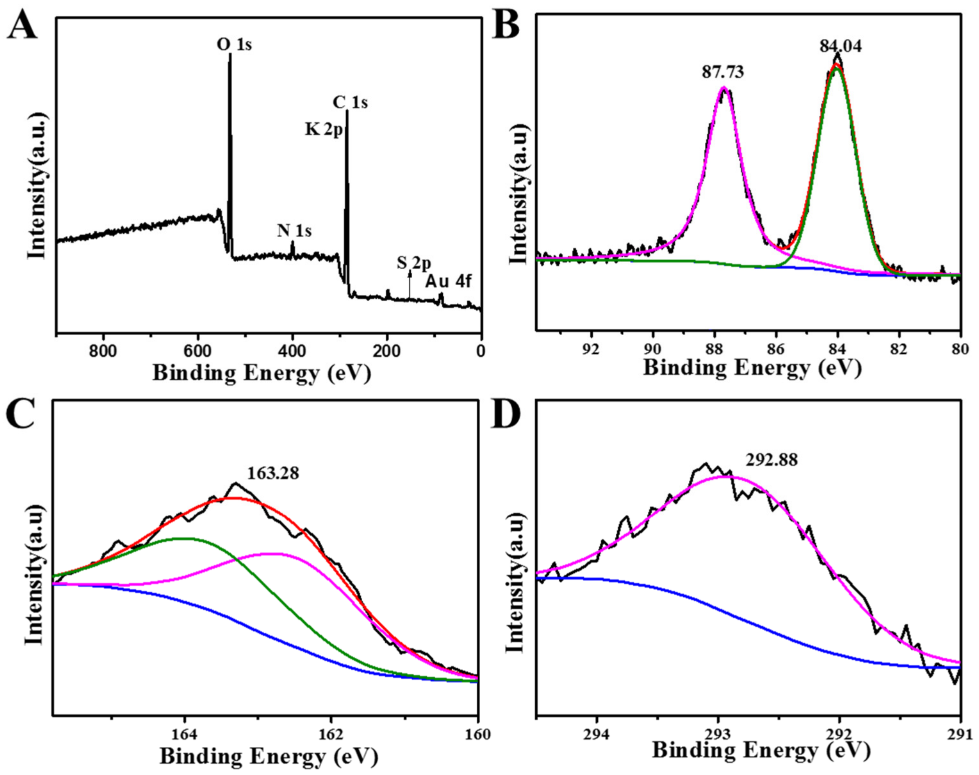

Figure S1 (A) XPS survey spectra of the as synthesized Au NCs/5-FU@Carr-LA; the high-resolution XPS spectra of (B) Au 4f; (C) S 2p; (D) K 2p.

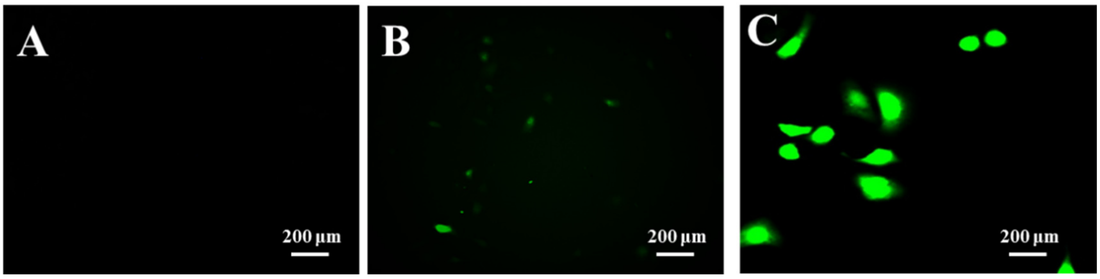

Figure S2 CLSM images of FITC-dextran labeled (A) HepG2 cells, (B) Au NCs/5-FU@Carr and (C) Au NCs/5-FU@Carr-LA.

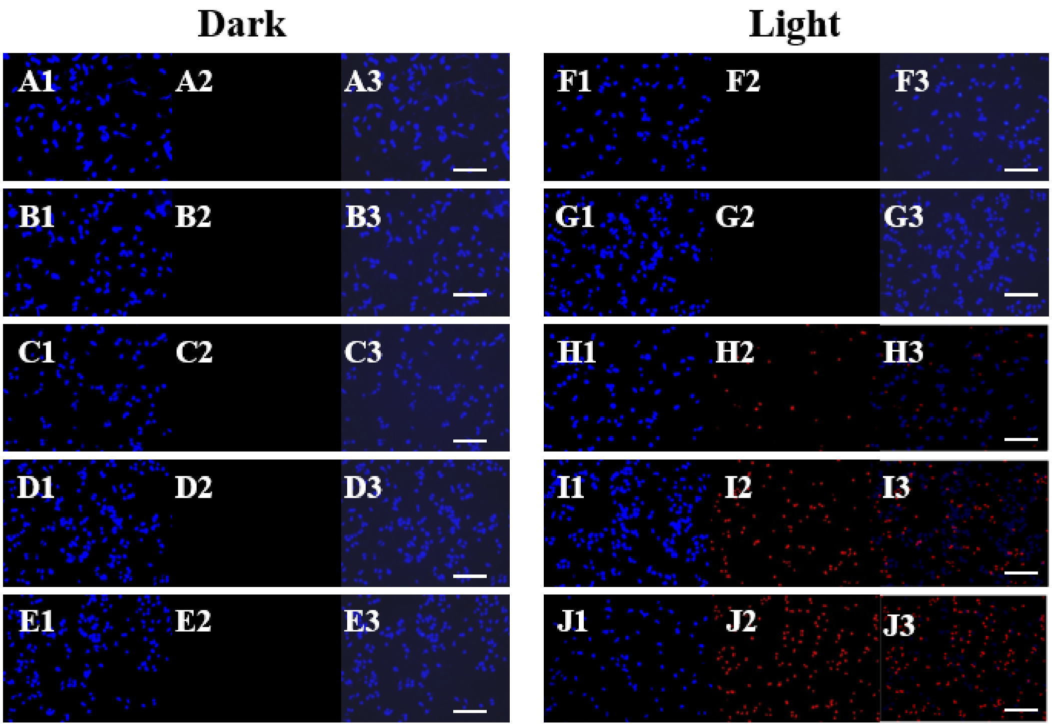

Figure S3 Fluorescence images of HepG2 cells incubated with (A, F) Control (without samples), (B, G) Carr, (C, H) Au NCs@Carr, (D, I) Au NCs/5-FU@Carr, (E, J) Au NCs/5-FU@Carr-LA without laser irradiation (A, B, C, D, E) or with laser irradiation (F, G, H, I, J).

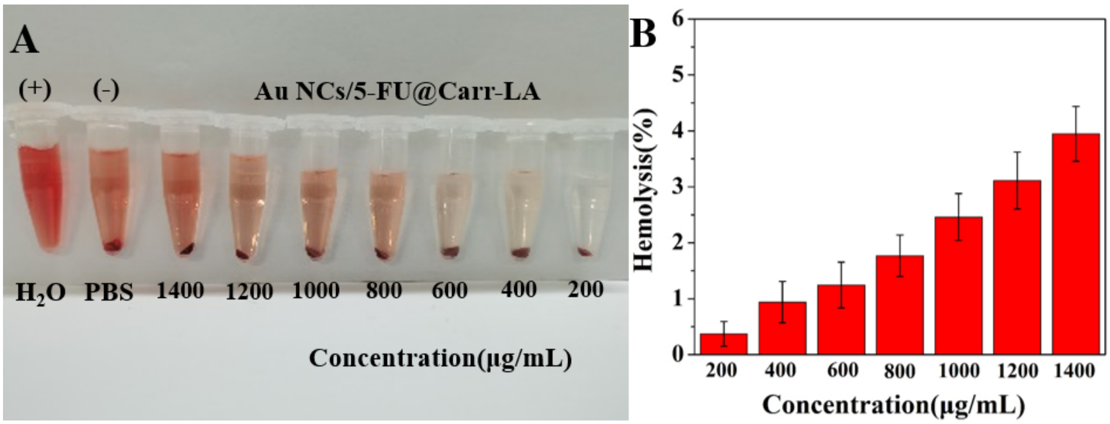

Figure S4 (A) The hemolysis percentage in the presence of the Au NCs/5-FU@Carr-LA at different concentrations and (B) The hemolysis photographs.

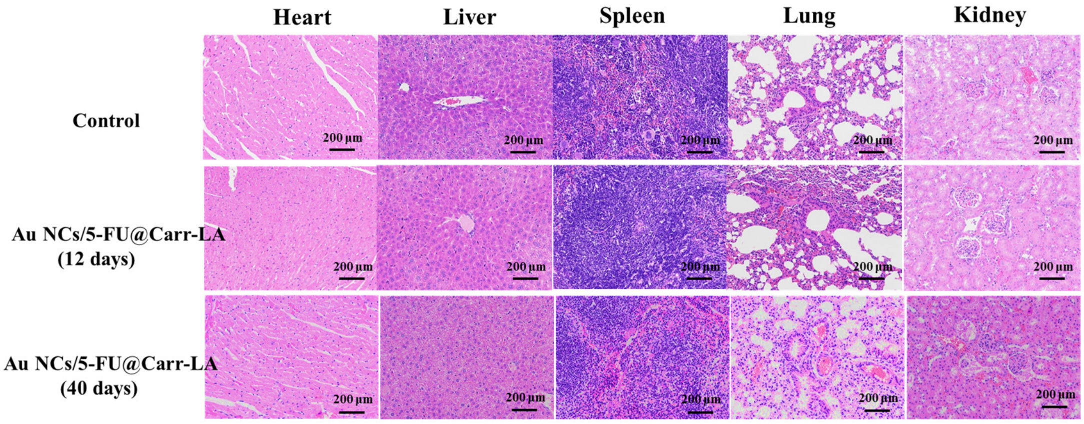

Figure S5 The H&E staining of mice main organs after Au NCs/5-FU@Carr-LA treatments for 12 and 40 days. (Scale bar: 200 μm).

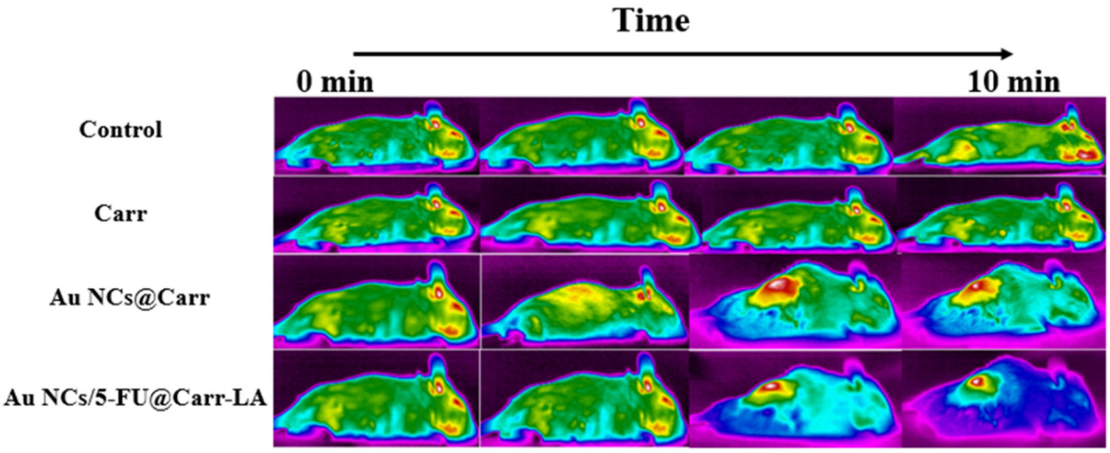

Figure S6 The corresponding IR images of ICR tumor-bearing mice treated with control (top) and Au NCs/5-FU@Carr-LA (bottom) under irradiation at varied time intervals (0, 2, 4, 6, 8, and 10 min).
